# Mapping activity of grazing cattle using commercial virtual fencing technology

**DOI:** 10.3389/fvets.2025.1536977

**Published:** 2025-03-12

**Authors:** Kareemah Chopra, Tom Craig Cameron, Roger C. Beecroft, Luke Bristow, Edward A. Codling

**Affiliations:** ^1^School of Mathematics, Statistics and Actuarial Science, University of Essex, Colchester, United Kingdom; ^2^School of Life Sciences, University of Essex, Colchester, United Kingdom; ^3^Red Cow Ltd., Suffolk, United Kingdom; ^4^Legacy Grazing, Chelmsford County Hall, Chelmsford, United Kingdom

**Keywords:** cattle (*Bos taurus*), grazing, space-use, virtual fencing, Brownian bridge movement models

## Abstract

Identifying where and how grazing animals are active is crucial for informed decision-making in livestock and conservation management. Virtual fencing systems, which use animal-mounted location tracking sensors to automatically monitor and manage the movement and space-use of livestock, are increasingly being used to control grazing as part of Precision Livestock Farming (PLF) approaches. The sensors used in virtual fencing systems are often able to capture additional information beyond animal location, including activity levels and environmental information such as temperature, but this additional data is not always made available to the end user in an interpretable form. In this study we demonstrate how a commercial virtual fencing system (Nofence®) can be used to map the spatiotemporal distribution of livestock activity levels in the context of grazing. We first demonstrate how Nofence® activity index measurements correlate strongly with direct in-situ observations of grazing intensity by individual cattle. Using methods adapted from movement ecology for analysis of home range, we subsequently demonstrate how space-use and cumulative and average activity levels of grazing cattle can be spatially mapped and analyzed over time using two different approaches: a simple but computationally efficient cell-count method and a novel adapted version of a more complex Brownian Bridge Movement Model. We further highlight how the same sensors can also be used to map spatiotemporal variations in temperature. This study highlights how data generated from virtual fencing systems could provide valuable additional insights for livestock managers, potentially leading to improved production efficiencies or conservation outcomes.

## 1 Introduction

Livestock grazing is a widespread agricultural practice with profound implications for ecosystems, land management and production efficiency (1). To balance livestock needs and pasture quality, grazing must be carefully managed, as overgrazing can deplete plant and soil nutrients (2). Methods such as rotational grazing, where livestock are periodically moved between areas of a pasture to allow plant regrowth (3), and “mob grazing,” involving short duration, high-density grazing followed by a grass recovery period (4), can improve herbage availability (5–7). However, these systems require careful management, as poor timing can deplete forage quality and livestock performance (8, 9). Carefully managing livestock density and grazing behavior can also support conservation aims, including improved biodiversity and habitat quality (10, 11).

Identifying the location, intensity and frequency of grazing is crucial for informed livestock and conservation management decision-making (12–14). Traditional monitoring methods, such as visual observation and field surveys are labor- and time-intensive (15, 16). The introduction and uptake of Precision Livestock Farming (PLF) approaches have increased the use of automated monitoring technologies that continuously monitor livestock, such as Global Navigation Satellite Systems (GNSS) that track location (17–19) and accelerometers that record activity (20, 21). In wild animals, ranging from fish to mammals, advances in sensor technology and the availability of large movement datasets have led to the development of sophisticated analysis methods within the field of movement ecology, the study of the movement patterns of animals in relation to their environment (22, 23). Tools such as random walk movement models (24), Hidden Markov Models (HMM) (25), State-space Models (SSM) (26), and Brownian Bridge Movement Models (BBMM) (27), have the potential to provide deeper insights into the movement and space-use behavior of managed animals (28, 29). Previous studies have identified grazing behavior using accelerometers (30, 31), but linking activity data to location data can help pinpoint where livestock are grazing (32–34) or where barn-housed animals may be feeding (28, 35).

Virtual fencing systems typically consist of animal-mounted collars that use GNSS signals to monitor individual livestock location and give an audio cue or mild electrical impulse once an animal crosses a user-defined virtual boundary. The end-user sets the virtual boundary using a smartphone or computer application that enables basic visualization of position and other relevant data; commercially available virtual fencing systems include Vence® (United States); eShepherd® (Australia) and Halter® (New Zealand) (36). In this study, we focus on Nofence® (37), a system commonly used in the United Kingdom and Europe (38, 39). The Nofence® system records animal location and a relative activity index but does not directly inform end-users where cattle are actively grazing, although several recent studies have explored this potential. Hamidi et al. (40) used Nofence® GNSS location data to classify cattle as either lying (corresponding to at least two successive minutes without a movement relocation) or not lying (daylight hours spent not lying), and then combined these with Unmanned-Aerial-Vehicle (UAV) imaging to estimate grazing impact using the Red-Green-Blue Vegetation Index (RGBVI). Aaser et al. (39) used Nofence® location and activity index data to estimate grazing habitat preference. Using standard default system settings, they characterized a high activity index as grazing and a low activity index as not grazing. However, their study lacked ground-truth validation of grazing classifications, and highlighted the need for further work with real in-situ observations. Versluijs et al. (41) undertook in-situ observations to identify and classify various free-ranging cattle behaviors including grazing, but used high-resolution 10 Hz accelerometer measurements under a non-standard system configuration, which would lead to high battery drain therefore limiting long-term practical use (39).

In this study, we highlight how the activity index recorded by the Nofence® collar strongly correlates with direct in-situ observations of grazing intensity. Using data obtained from the Nofence® collar, we further demonstrate how tools adapted from the field of movement ecology can be used to visualize and map space-use, cumulative and mean activity intensity, and also localized temperature across the study site over time. We discuss how such data analysis could provide valuable additional insights for farmers and graziers using virtual fencing systems to manage their livestock.

## 2 Methods

### 2.1 Study site and duration

**Figure 1:**
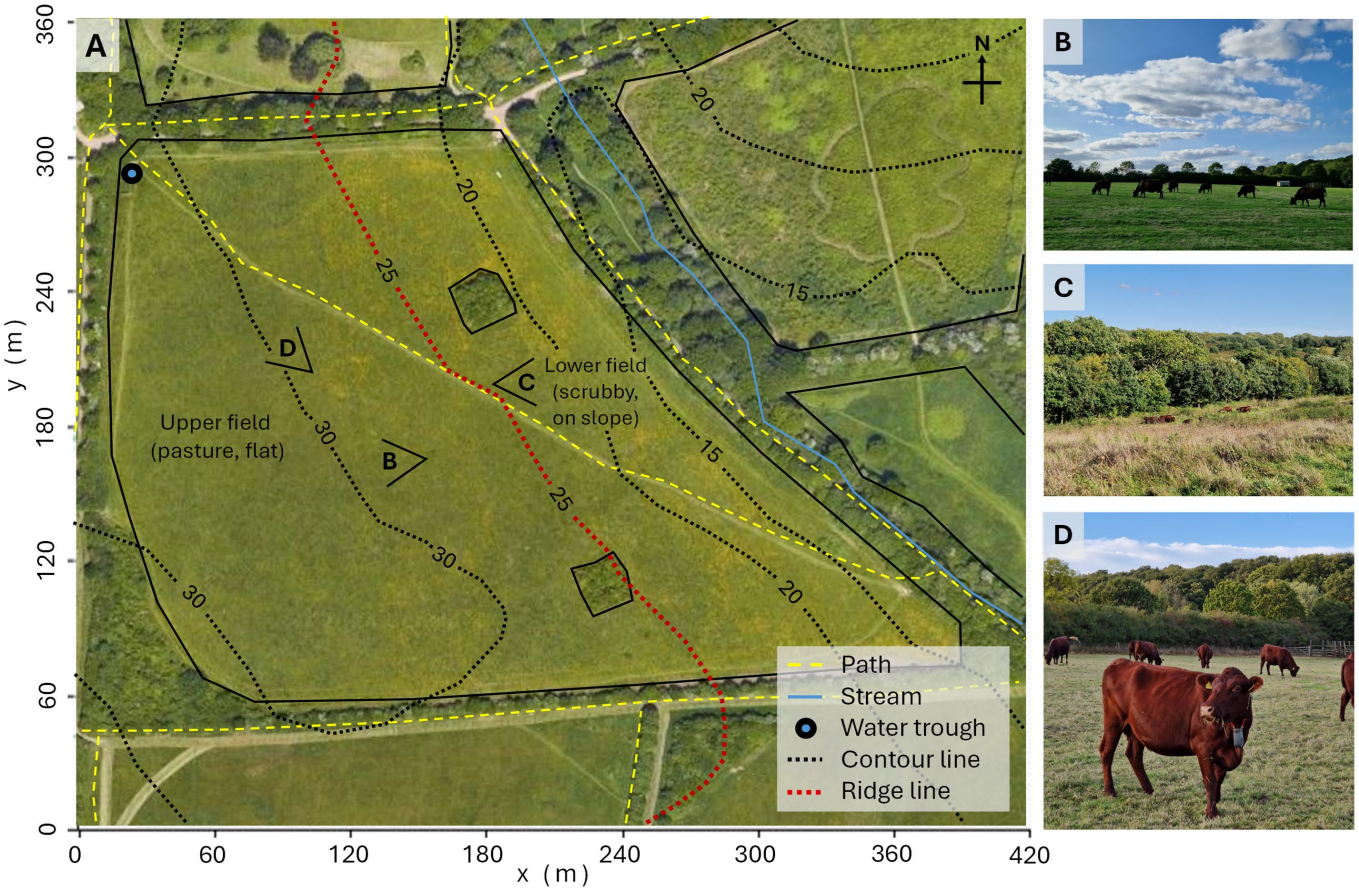
**(A)** Top-down map of the study site Boat Field, Highwoods Country Park, Colchester, Essex, UK; underlying image taken from Google Maps (43). Solid black lines mark field boundaries and fenced tree areas; dashed yellow lines indicate paths, the blue line shows a stream outside the field; a water trough is marked in the NW corner (blue circle with black border). Dotted black lines represent 5 m contour lines (height specified in meters) with the ridgeline dividing the upper and the lower fields marked in red line. Photo viewpoints are shown with a ‘V’ icon of the: **(B)** upper field (relatively flat pasture), **(C)** lower field (sloped, with scrubby vegetation), and **(D)** study cattle equipped with the Nofence® collar units (in the upper field).

The study was conducted from 29 September 2023 to 26 November 2023 (58 days) at Boat Field, High Woods Country Park, Colchester, Essex (postcode CO4 5WF; central Global Positioning System (GPS) coordinates of 51.902883, 0.910765). The field is a managed pasture, measuring approximately 7.2 hectares and 350 m x 300 m at its greatest extent (longitude x latitude), and consists of an upper, relatively flat section with short, hay-cut grass (Figures 1B,D), and a lower, sloped region with scrubby vegetation and small fenced areas of trees (Figures 1A,C; Supplementary material S1 provides further details, including plant species present). These two distinct areas were separated by a clear ridgeline (marked as the red contour line in Figure 1A), but livestock could move freely around the full field. Key features at the study site included paths, a stream outside the field, and a water trough in the north-west corner (Figure 1). No public access was permitted to the field during the study period.

### 2.2 Animals and sensors

Ethical approval (reference number ETH2324-0100) was provided by the University of Essex ethics committee. Using a herd of Red Poll cattle (*Bos taurus*), the study site was managed for conservation purposes on behalf of Colchester City Council by Legacy Grazing (authors RB and LB). On 29 September, Nofence® tracking collars were fitted to three cows (Figure 1D; cattle IDs 294,322, 294,361, and 294,364). These cows were all pregnant and suckling calves during the study, and formed part of a larger herd that varied in size over time (see Supplementary material S1 for further details).

The Nofence® tracking collar units include a GPS sensor, a dynamometer and an accelerometer to measure activity, and a temperature sensor (39, 42). End-users can set a virtual fence to constrain livestock via the collar units using a smartphone application (38, 39). In this study, the virtual fence was set approximately 60 m outside the boundary of the field, which was enclosed by a robust wooden fence, so was effectively unused given our aim was to use the collars to map space-use and activity, rather than specifically explore the impact of the virtual fence on behavior. Default collar settings were used for recording frequency as in Aaser et al. (39), with GPS locations recorded every 15 min when animals were active. To conserve battery, a lower update rate of approximately one recording every 1–2 h is used when animals are inactive, such as at night. Activity was measured using an in-built dynamometer, that counted movements along the heave axis over a defined (commercially sensitive) threshold, cumulatively totaled every 30 min and termed an ‘activity index’ by Aaser et al. (39). The collar unit also recorded the localized instantaneous temperature every 30 min. This dataset is available in the University of Essex Data Repository (44).

### 2.3 Grazing observations

The three collared cows were observed in-person by two observers over 8 days in October and November 2023. Each cow was continuously observed for an average of 936 min (minimum = 886 min, maximum = 1,035 min). Behavior was recorded as grazing when the cow was observed with their head lowered, actively consuming vegetation and not ruminating (Supplementary material S2); minor pauses in grazing (e.g., raising head and chewing for a few seconds) were noted but the classification was only changed to non-grazing (or vice-versa) when a clear and sustained change in behavior was observed. This observational data is available in the University of Essex Data Repository (45). All observation sessions were filmed using a video camera to enable validation of behavioral classification. For subsequent analysis, each minute throughout the study period was classified as belonging to a grazing or non-grazing bout, and the number of minutes (proportion of time) spent grazing (or non-grazing) in each 30-min observation period was determined.

To assess the correlation between the Nofence® activity index and observed grazing intensity, a linear model was used to compare the proportion of time each cow was observed to be grazing over each 30-min period to the sensor-recorded activity index from the same period. Model assumptions (linearity, normality, homoscedasticity, and independence) were checked and met. Supplementary material S2 provides further details of the observations and shows no significant difference between observers in the proportion of time observed grazing and activity index (e = −6.13, *p* = 0.15).

### 2.4 Mapping spatial distributions

Two methods were used to generate utility distribution (UD; density) maps of space-use (total time spent in given locations), cumulative activity (total sum of activity index), and average activity (mean activity index). The cell count method is computationally efficient but limited by data frequency, while the Brownian Bridge Movement Model (BBMM) estimates movement between recorded locations but it is more computationally intensive and requires model assumptions. For each spatial distribution generated, core and full range sizes were determined by identifying the highest density cells cumulatively adding to 50% or 95% respectively, following previous animal home range analyses [e.g. (28, 46, 47)]. All analyses were programmed using R Statistical Software v4.2.3 with R Studio Cherry Blossom (48, 49), employing the ‘ggplot2’ and ‘ggmap’ (function ggmap()) packages (50, 51) for plotting distribution matrices over satellite imagery.

In the cell count method, a virtual grid (15 m x 15 m = 225 m^2^ cells) was overlayed over the field layout (28). At each time-step, GPS data were used to assign each individual cow to a given cell and the respective cell count value was increased to reflect time spent in the area (based on time duration between 15-min location recordings), cumulative activity (recorded every 30 min and split equally between consecutive locations), average activity or average sensor temperature. A BBMM (27) was used to estimate movement paths between recorded locations and hence generate space-use and activity maps within the same 15 m x 15 m grid, although this required a novel modification to the standard BBMM algorithm in order to map activity across space. Where animals are relatively stationary, the cell count method and the BBMM yield similar results, but the BBMM may give better estimates when movement is more dynamic. Supplementary material S3 provides full details of these spatial mapping methods and their implementation.

Sensor recorded temperatures, influenced by the close proximity of the collar unit to the animal (see Figure 1D; Supplementary material S2), will not directly measure ambient conditions but may nevertheless reflect underlying trends. To demonstrate the relation between hourly sensor-recorded temperatures and hourly local weather station temperature readings (within a mile of the study site, 52), a Spearman’s Correlation test was conducted (non-normal data distribution, Shapiro-Wilks = 0.99, *p* < 0.001).

#### 2.4.1 Comparing the similarity of spatial distributions

The Bhattacharyya coefficient (BC) measures the similarity between two probability distributions. After normalizing the spatial distributions, to be consistent with a probability density, BC was used to compare the cumulative activity, average activity, and space-use across all three cattle (Equation 1):


(1) $$BC=\sum \left(\sqrt{P_{i}\times Q_{i}}\right)\text{,}$$

where *P_i_* and *Q_i_* are the normalized values of the distributions, and the sum is taken over all grid cells. A higher BC indicates a greater similarity, with 0 indicating no similarity and 1 indicating complete similarity.

The BC was also used to compare the recorded temperature to space-use and activity, and to compare space-use and activity over time and between each cow (see Supplementary material S3).

## 3 Results

### 3.1 Comparison of sensor-recorded activity index and observed grazing

**Figure 2:**
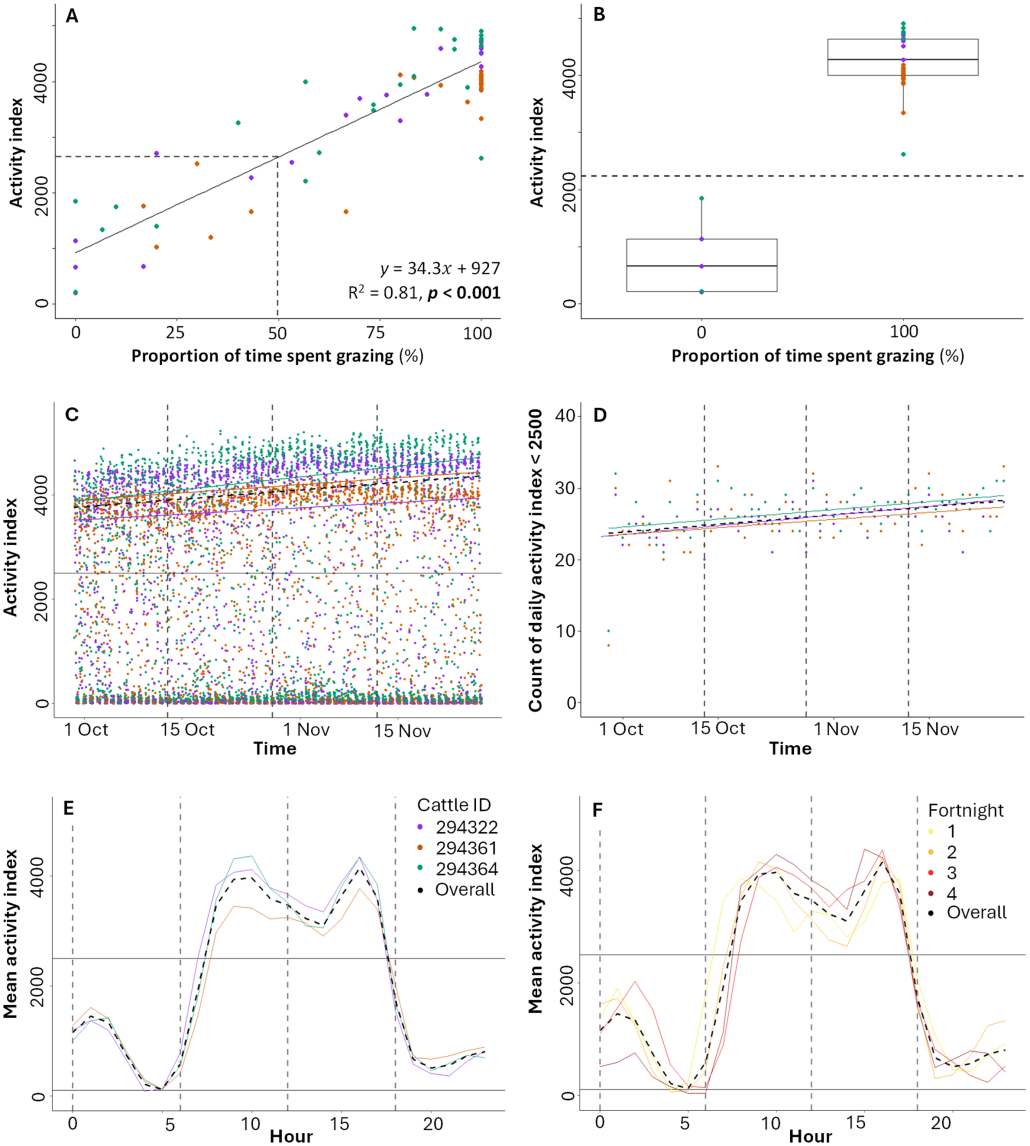
**(A)** Activity index in relation to proportion of time spent grazing, and **(B)** comparison of activity index during non-grazing periods (proportion of time spent grazing = 0%; *n* = five data points) and grazing periods (proportion of time spent grazing = 100%; *n* = 31 data points). Each data point represents a 30-min observation period (and associated sensor activity index measurement). **(C)** Activity index over time, with each data point representing a 30-min sensor-recorded period. Lines show overall and individual trendlines for activity index >2,500. **(D)** Daily count of activity index values <2,500 (low intensity grazing) over time, with overall and individual trendlines. **(E)** Mean hourly average activity index over the full study for all three cattle combined and individually. Dashed vertical grey lines mark divisions by time of day: night (00:00:00 to 05:59:59), morning (06:00:00 to 11:59:59), afternoon (12:00:00 to 17:59:59) and evening (18:00:00 to 23:59:59). **(F)** Mean hourly average activity index for each two-week period and for the overall study, for all three cattle combined. In **(A)** the dashed line marks the estimated activity index where the proportion of time spent grazing is 50% (activity index = 2,642). In **(B)**, the dashed lined shows activity index = 2,233, the midpoint between the maximum value where proportion of observed time observed grazing is 0% (activity index = 1848) and the minimum value where the proportion of observed time spent grazing is 100% (activity index = 2,617). In **(C,D)** dashed vertical grey lines show two-week divisions (F). In **(C–F)** the solid horizontal line corresponds to an activity index of 2,500. Overall trendlines are shown in each subplot (dashed black).

The mean observed time for a grazing bout was 20 min 42 s and for a non-grazing bout was 10 min 42 s. A strong significant positive relationship exists between the relative proportion of time cattle were observed grazing over a 30-min period and the activity index recorded by the Nofence® sensor (*R*^2^ = 0.81, *p* < 0.001; Figure 2A). Cattle were primarily observed during daytime grazing, resulting in more exclusively grazing periods (*n* = 31 periods) than exclusively non-grazing periods (*n* = 5 periods), with *n* = 36 mixed periods where both grazing and non-grazing behaviors occurred (Figure 2B). During exclusively grazing periods, the activity index is clearly higher (100% grazing over 30 min, median activity index = 4,270) compared to periods of exclusively non-grazing (0% grazing, median activity index = 661) (Figure 2B). Non-zero activity index values were recorded during exclusively non-grazing behavior (Figure 2B), likely due to other head movements such as drinking, scratching, ruminating, or interactions between cows. Results remained consistent when considering data from each observer independently (see Supplementary material S2). For further analysis, and consistent with Aaser et al. (39), we classify activity as ‘low intensity’ when the activity index <2,500 and ‘high intensity’ when the activity index >2,500. This threshold is a heuristic estimate that approximately aligns with the observed proportion of time spent grazing being 50% (activity index = 2,642; Figure 2A) and the midpoint (activity index = 2,233; Figure 2B) between the maximum value for exclusively non-grazing periods (activity index = 1,848) and the minimum value for exclusively grazing periods (activity index = 2,617).

### 3.2 Activity over time

High intensity activity periods (activity index ≥2,500) account for 45% of the sensor recorded activity index values (Figures 2C,D), with a gradual increase in their daily occurrence from October to December (Figure 2C). Across the three cattle, average activity during high intensity periods increased over time (Spearman’s Correlation Coefficient [hereafter *ρ*] = 0.18, *p* < 0.001; values above black line in Figure 2C), but average activity during low intensity periods (activity index <2,500) decreased over time (*ρ* = −0.08, *p* < 0.001; values below black line in Figure 2C). The daily count of low intensity periods increased over time (Figure 2D; *ρ* = 0.37, *p <* 0.001), despite the increase in average activity during high intensity periods over the same period (Figure 2C).

A consistent diurnal activity pattern is shown across all cows, with the mean hourly activity index increasing from low intensity activity overnight (mean activity index <2,500 from 18:00 to 07:00) to high intensity activity in the late morning and early afternoon, with a slight dip around midday, peaking at 10:00 and 16:00 (mean activity index = 3,963 and 4,152 respectively), followed by a decrease into the evening and night (Figure 2E). Mean hourly activity index values are similar for each individual cow, although some differences are apparent (e.g., cattle ID 294361 consistently showed lower mean activity during the day compared to the other cows; Figure 2E). The mean hourly activity index also increased over the study period (by two-week periods; Figure 2F), aligning with the increase in high intensity grazing in Figure 2C.

### 3.3 Mapping space-use and grazing activity

**Figure 3:**
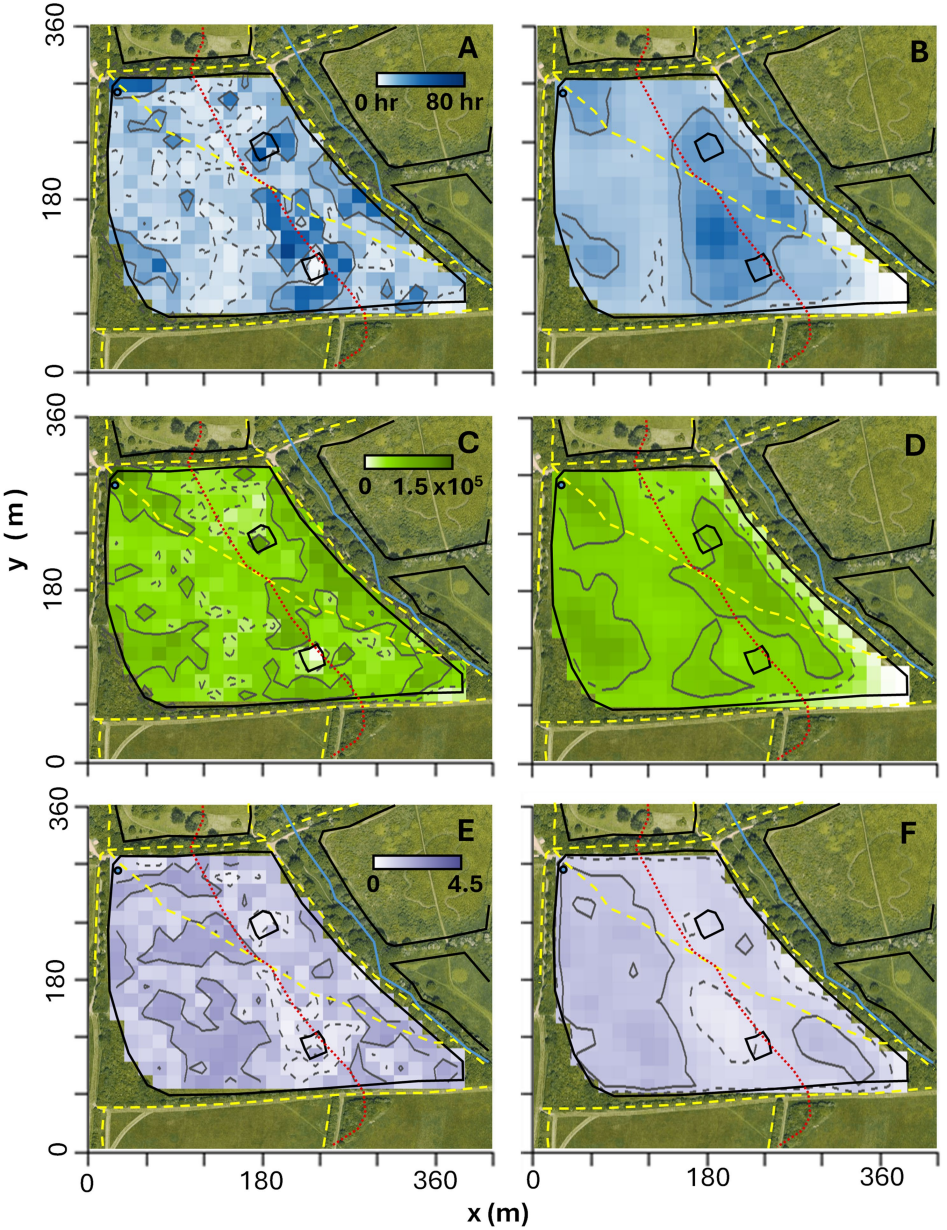
Distribution maps generated using the **(A,C,E)** cell count method and **(B,D,F)** Brownian Bridge Movement Model (BBMM): **(A,B)** space-use, **(C,D)** cumulative activity and **(E,F)** average activity over the full study and all three cattle. Each virtual cell is 225 m^2^ and darker colors indicate higher activity, while lighter colors indicate lower activity. Contours represent core range (50%; solid grey) and the full range (95%; dashed grey). The red dashed line shows the contours of the ridgeline dividing the upper and the lower fields. Note that there are no units for the density scales in **(C-F)** as the Nofence^®^ activity index is used, which is a dimensionless count.

The cell count method and the BBMM produced qualitatively similar results for space-use across the study (Figures 3A,B), with the BBMM generating a smoother map due to the model estimation of movement in-between actual observed locations. Both models consistently identified core range areas near the water trough (NW corner of upper field), the SW corner of the upper field, along the ridgeline and in the lower field (Figures 3A,B).

Comparing Figures 3A,C for the cell count method, and Figures 3B,D for BBMM, highlights that space-use is highly correlated with cumulative activity (BC = 0.96 for cell count method; BC = 0.99 for BBMM). Nevertheless, there are some notable differences in spatial distribution such as the upper field where the core range for cumulative activity is larger (Figure 3C, core range in upper field = 75 cells for cell count method; Figure 3B, core range in upper field = 54 cells for BBMM) than for space-use (Figure 3A, core range in upper field = 33 cells for cell count method; Figure 3B, core range in upper field = 47 cells for BBMM). The (mean) average activity distributions show larger core range in the upper field (Figure 3E, core range = 79 cells in upper field and 25 cells in lower field for cell count method; Figure 3F, core range = 99 cells in upper field and 29 cells in lower field for BBMM), indicating that there may be more spatial uniformity in average activity across the field when compared to cumulative activity (Figures 3C,D), although the distribution of average activity intensity does not directly overlap with cumulative activity (BC = 0.93 for the cell count method, BC = 0.93 for BBMM).

Supplementary material S3 includes more detailed analysis of spatiotemporal variations in space-use and activity. In particular, Supplementary Figures S3.3–S3.6, respectively, highlight how the cattle shifted their space-use and activity from the lower field at night to the upper field during the day, with a further consistent shift from the upper to lower field as the study progressed.

### 3.4 Temperature

**Figure 4:**
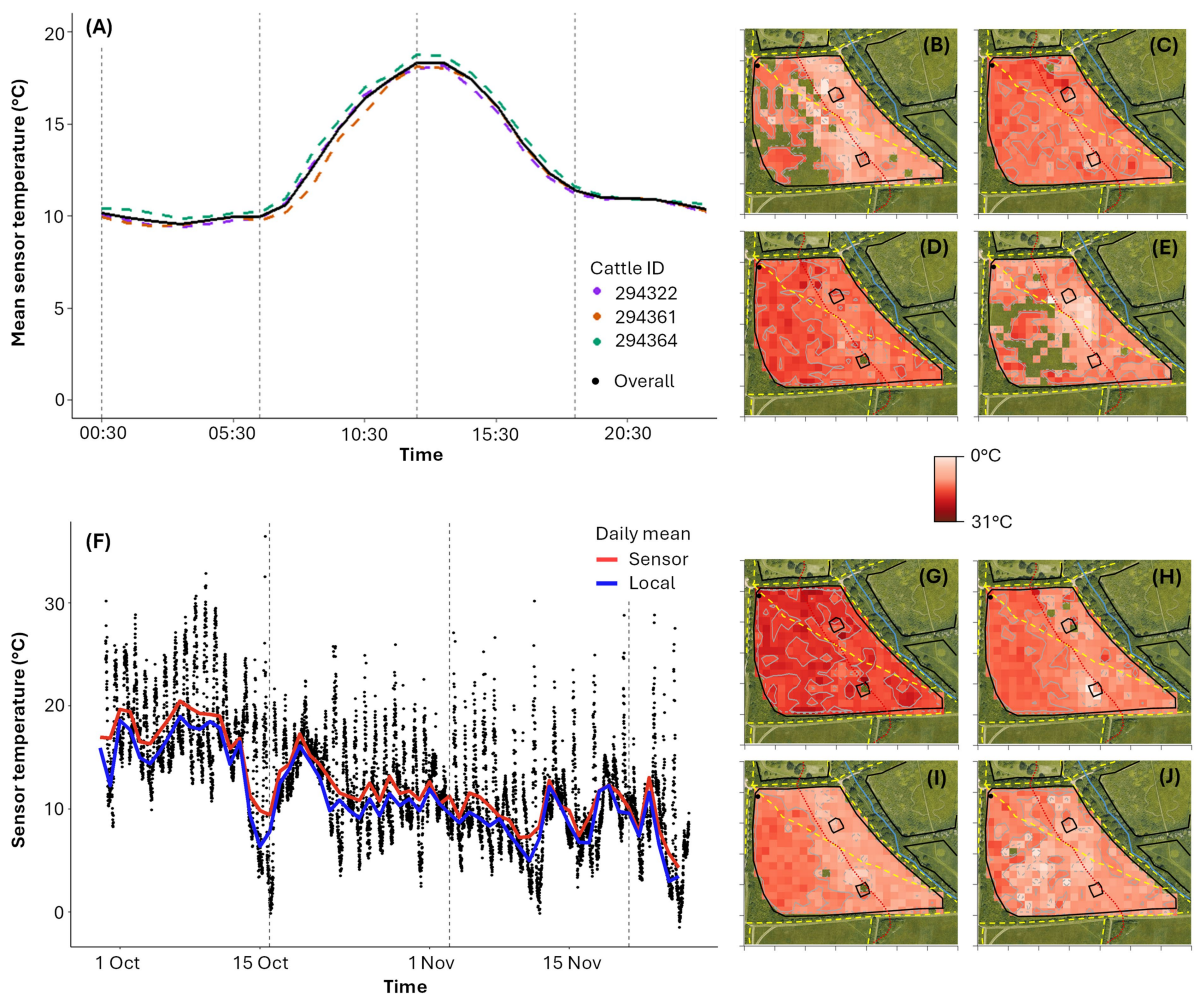
**(A)** Average sensor temperature per hour of day over the full study duration. Hourly sensor temperatures are calculated as the mean of temperatures recorded between 00:00 and 00:59, 01:00 to 01:59, 02:00 to 02:59 etc. Dashed vertical grey lines show divisions by time of day: night (00:00:00 to 05:59:59), morning (06:00:00 to 11:59:59), afternoon (12:00:00 to 17:59:59) and evening (18:00:00 to 23:59:59). **(B–E)** Temperature distribution maps by time of day across all three cattle: **(B)** night (*n* = 2090 data points), **(C)** morning (*n* = 2088 data points), **(D)** afternoon (*n* = 2,115 data points), **(E)** evening (*n* = 2,124 data points). **(F)** Sensor temperature over the study duration from all three animal-mounted sensors, with a mean daily trendline shown in red. A mean daily trendline is also shown for local weather station temperature, less than a mile from the study site, in blue. Dashed vertical grey lines show two-week divisions. **(G–J)** Temperature distribution maps for each two-week period: **(G)** F1 (*n* = 1937 data points), **(H)** F2 (*n* = 2,304 data points), **(I)** F3 (*n* = 2,160 data points) and **(J)** F4 (*n* = 2016 data points). In **(B–E)** and **(G–J)**, each virtual cell is 225m^2^, with darker red indicating higher temperatures and white indicating lower temperatures (0°C to 31°C). The cells in **(B–E)** and **(G–J)** with a satellite underlay show areas with no recorded data. The red dashed line represents the contour of the ridgeline dividing the upper and the lower fields.

As expected for this temperate location, mean hourly temperature exhibits a consistent diurnal pattern, peaking at 18.33°C (13.00–13:59) and reaching a minimum of 9.58°C (03:00–03:59) over the study period, with similar patterns in all cow sensors (maximum difference of 1.79°C for 07:00–07:59; Figure 4A). This diurnal pattern is also clear in the spatial temperature distribution maps, showing higher temperatures in the morning and afternoon (Figures 4C,D) compared to the night and evening (Figures 4B,E). There is a significant strong correlation between the sensor recorded temperatures and local weather station temperature readings (*ρ* = 0.88, *p* < 0.001), although the sensor temperature measurements were consistently slightly higher, likely due to the proximity between the animal and the collar (Figure 4F). Although the relative variation in mean temperature across the field is small (coefficient of variation = 20.66%, assuming no spatial dependence between cells), the lowest recorded temperatures occur around the ridgeline (Figures 4B–E), possibly due to increased wind exposure which was generally from the SW during the study period. Over the study period, temperatures gradually decreased (Figure 4F), with two-week spatial temperature maps illustrating the first 2 weeks were notably warmer (mean temperature of 18.23°C) than later periods (mean temperatures of 12.55°C, 10.11°C and 9.57°C for F2 to F4 respectively; Figures 4F,G).

## 4 Discussion

This study has highlighted how data routinely recorded by a commercial virtual fencing technology can be used to provide additional insights into the space-use and activity of cattle. Activity index measurements recorded by the Nofence® tracking sensor were shown to be highly correlated with grazing intensity, as determined and validated by direct in-situ observations of cattle in a United Kingdom pasture. Two different approaches adapted from movement ecology, a simple cell count and a more complex Brownian Bridge Movement Model (BBMM), were used to map space-use and activity intensity over time and space. These approaches can provide additional insights and information for farmers and graziers managing livestock using this type of technology.

The strong significant positive relationship between the Nofence® activity index and the proportion of time cattle were directly and continuously observed grazing over 30-min periods (Figure 2A) highlights how the activity index could offer a useful indirect proxy measure of relative grazing intensity. This measure could be continuously collected over time and space using default commercial system settings. Previous studies using the same system have either lacked validation of the activity index against grazing observations (39), have indirectly estimated grazing intensity through location data alone (40), or have used high-frequency accelerometry data which is unavailable in the standard commercial system and may also limit battery life (41).

Monitoring patterns of activity over time could offer insights into grazing behavior to help inform managers. Grazing cattle are generally more active during the daytime (39, 53, 54) and consistent diurnal patterns in the activity index were observed for all cattle in the study (Figure 2E). Both overall mean activity index (Figure 2F) and the mean activity index during high intensity activity periods (Figure 3C) increased over the study, while counter-intuitively, the daily count of low intensity activity periods also increased (Figure 2D). This suggests that, as the study progressed, cattle spent less total time in the day grazing but were more active when doing so, and likely reflects changes in the availability of forage (55, 56) and reduced daylight hours (53, 54) toward the end of the year, which are both known to affect cattle grazing behavior.

The cell count (28, 57) and BBMM (27, 58) methods are standard approaches in movement ecology to analyze animal space-use over time (Supplementary material S3), although alternative statistical methods such as Hidden Markov Models [HMMs (25, 59, 60)] and State Space Models [SMMs (26, 61, 62)] could also be used to identify behavioral changes over space and time. The cell count method is efficient for small areas with high frequency data but does not account for movement between recorded locations. The BBMM offers a more complete interpretation of movement but is computationally intensive and requires model parameter estimation and assumptions. Both methods yielded qualitatively similar space-use maps (Figures 3A,B), though the BBMM produces smoother visualizations. The choice of grid resolution and location recording frequency are critical for both methods, which may break down when data are sparse (Supplementary material S3). Clear space-use patterns emerged throughout the study, with cattle consistently positioned near the water trough, along the ridgeline, and in areas such as the South-West corner of the upper field (Figures 3A,B). Previous studies have shown how grazing patterns may link to vegetation (63–65), external resources (66–68) and stocking rate (69, 70), but more detailed environmental data would be required to fully explain cattle space-use in this study. Similarly, a sample size of three is too small to properly analyze inter-individual differences in space-use but there is some evidence that individual cows exhibited distinct preferences for certain areas of the field (Supplementary material S3).

By modifying the standard BBMM algorithm to incorporate recorded activity index values in a stepwise manner (Supplementary material S3), we can spatially map both cumulative and average activity (Figures 3C–F). Space-use distribution maps correlated closely with cumulative activity maps (cell count method, see Figures 3A,C and BBMM, see Figures 3B,D), indicating that cows were generally active in areas they frequented, especially the upper field. The cattle were often directly observed to lie and rest toward the South of the upper field near the ridgeline, explaining why space-use and average activity core range do not correlate there (most clearly seen by comparing Figures 3B,F). While average activity was generally higher in the upper field (Figures 3E,F), it did not always coincide with the highest cumulative activity areas (Figures 3C,D). This suggests that some less frequented areas may experience intensive short-term activity (grazing) over short periods. Spatial variation in cattle grazing activity has been observed to be driven by vegetation preferences (71, 72) or by time of day (39); Supplementary Figures S3.3, S3.4 in Supplementary material S3 highlight how activity changed over space over the course of each day. The long-term distribution of cattle activity may also fluctuate due to changes in the availability of vegetation (56, 73) or water sources (72, 74); a decrease in the quantity and availability of vegetation in the upper field may explain a notable shift toward the lower field as the study progressed (Supplementary Figures S3.5, S3.6 in Supplementary material S3).

Mean sensor-recorded temperature showed a diurnal pattern (Figure 4A) and decreased over the study (Figure 4F), consistent with seasonal trends in the United Kingdom (75). Although temperature variations across the field were minimal (CV = 20.66%), lower temperatures were recorded on or near the ridgeline (Figures 4B–E). Sensor temperatures are generally higher than local weather station temperatures, most likely due to the sensor’s close proximity to the animal (Supplementary material S2). Using an animal-mounted sensor to record temperature and other environmental data could offer novel ways to understand and interpret both localized climatic conditions and related animal behavior. For example, when precipitation increases, cattle may expand their foraging area (74) or roam further (76), and in hot weather, cattle may graze away from open pasture where shade may be lacking (67). However, there are some limitations to this ‘mobile sensor’ approach since observations are inherently biased toward areas where the animal spends more time, and certain locations may miss data entirely at key times (e.g., Figures 4B,E show missing data in the upper field because cows tended to rest in the lower field overnight).

Using either of the methods highlighted in this study, long-term spatiotemporal activity monitoring could be undertaken using data recorded as part of the normal operation of virtual fencing systems. The cell count method is extremely simple and computationally efficient and hence could easily be incorporated in real time into standard visualization tools used as part of virtual fencing systems. The BBMM is more complex and may require additional computational resources but can model behavior in between recorded locations and produces smoother distribution maps to help end-users visualize outputs. Using outputs from either approach could help inform and improve grazing practices by guiding rotation frequency to maintain forage quality and prevent overuse (8, 9), or alert graziers to animal health and welfare concerns where significant activity or behavioral changes occur (29, 35, 77, 78), enabling timely interventions. In practical deployments of virtual fencing technology, boundary interactions that occur may not significantly affect overall cattle behavior (42, 79) but could alter space-use activity indices directly (e.g., animals moving quickly near the virtual boundary) or indirectly (e.g., animals behaving differently due to being constrained by the virtual fence). Hence better understanding of how the recorded activity index relates to virtual fence boundary interactions would be needed before our approach could be applied in contexts where the virtual fence is used as part of grazing management. Similarly, different local environments, varying forage availability, topography, and weather conditions, may lead to distinct patterns of activity and grazing behavior and hence further work will be needed to generalize this approach across landscapes and livestock types (80–82).

## Data Availability

The datasets generated and analysed during the current study are available in the University of Essex Data Repository, 10.5526/ERDR-00000215 and 10.5526/ERDR-00000216 (44, 45).
