# Supplementary material for: Mapping activity of grazing cattle using commercial virtual fencing technology
Source: Front Vet Sci. 2025 Mar 12;12:1536977. doi: 10.3389/fvets.2025.1536977 (PMC11960750; doi:10.3389/fvets.2025.1536977)
Supplement: Supplementary file 1 [file Data_Sheet_1.DOCX]

Supplementary Material 1: Study site, animals and sensors

This file provides further information on the study site’s environment (Section 1), the animals and sensors, including details on the cattle equipped with the Nofence® tracking collar units and key dates of herd changes (Section 2), as well as the technical specifications and performance of the collar units (Section 3).

# Study site

# Plant species were recorded in the field on 25th May 2023, and included common UK pasture species including grasses, legumes, herbs, shrubs and wildflowers (Table S1.1). Most of the larger shrubs were observed in the lower part of the field (refer to Figure 1 in the main paper), which also included some specific areas of fenced trees.

# Table S1.1. Plant species (Latin and common names) recorded at the study site, Boat Field, alongside their recorded frequency (frequent, locally abundant, occasional and rare).

| **Latin name** | **Common name** | **Recorded frequency** |
| --- | --- | --- |
| *Anthoxanthum odoratum* | Sweet vernal grass | Frequent |
| *Festuca rubra* | Red fescue | Frequent |
| *Galium verum* | Lady's bedstraw | Frequent |
| *Holcus lanatus* | Yorkshire fog | Frequent |
| *Lathyrus pratensis* | Meadow vetchling | Frequent |
| *Lotus corniculatus* | Common bird's-foot-trefoil | Frequent |
| *Ranunculus acris* | Meadow buttercup | Frequent |
| *Vicia sativa* | Common vetch | Frequent |
| *Crataegus monogyna* | Hawthorn | Locally abundant |
| *Rosa canina* | Dog-rose | Locally abundant |
| *Rubus fruticosus agg.* | Bramble | Locally abundant |
| *Achillea millefolium* | Yarrow | Occasional |
| *Briza media* | Quaking-grass | Occasional |
| *Centaurea nigra* | Common knapweed | Occasional |
| *Cirsium arvense* | Creeping thistle | Occasional |
| *Cynosurus cristatus* | Crested dog's-tail | Occasional |
| *Dactylis glomerata* | Cock's-foot | Occasional |
| *Lathyrus nissolia* | Grass vetchling | Occasional |
| *Plantago lanceolata* | Ribwort plantain | Occasional |
| *Ranunculus repens* | Creeping buttercup | Occasional |
| *Trifolium dubium* | Lesser trefoil | Occasional |
| *Vicia hirsuta* | Hairy tare | Occasional |
| *Daucus carota* | Wild carrot | Rare |
| *Knautia arvensis* | Field scabious | Rare |
| *Prunus spinosa* | Blackthorn | Rare |

# Animals and sensors

## From late September 2023, four cows (each with a calf) and one breeding bull grazed the study site. The cows grazed at a neighboring field prior to this. Cattle ID 294322 was born in December 2017 with a calf born February 2023, cattle ID 294361 was born in December 2018 with a calf born February 2023, cattle ID 294364 was born in March 2010 with a calf born in February 2023. On 1 October 2023 the bull was removed from the field and 12 heifers (born autumn 2021 to spring 2022) were delivered; on 28 October one (uncollared) cow and its calf and the 12 heifers were removed. The cows with tracking collars (and their calves) were removed on 6 December 2023. No additional feed was provided to the group during the study period except on the days when animals were delivered or removed (where cattle pellets were given to the individual animals being moved).

## Collar units

The Nofence® tracking collar unit is 858g in weight, 153.5mm x 145.4mm x 54.2mm (length x width x height). Outdoor mobile signal coverage was strong at the study site throughout the study (1). The collar unit operates on solar and battery energy with default settings over two months, the battery life dropped an average of 17% per sensor. Over the study, 15,332 location data points were recorded in total (from a theoretical maximum of 16,830 due to inactivity and occasional mobile coverage loss).

**References**

1. SignalChecker. Mobile Coverage Checker by postcode for all UK networks with one search. (2024) https://www.signalchecker.co.uk/ [Accessed May 3, 2024].
