# Supplementary material for: Mapping activity of grazing cattle using commercial virtual fencing technology
Source: Front Vet Sci. 2025 Mar 12;12:1536977. doi: 10.3389/fvets.2025.1536977 (PMC11960750; doi:10.3389/fvets.2025.1536977)
Supplement: Supplementary file 2 [file Data_Sheet_2.DOCX]

Supplementary Material 2: Grazing observations

Here, we provide detailed information on the in-person observations of the three cows collared with Nofence® collar units, including the specific observation sessions conducted by authors TC and EC. These observations were used to examine the relationship between grazing intensity and the activity index recorded by the Nofence® sensors (see Section 2.3 in main paper), and here considering observer influence.

The three collared cows were observed at the following times by two observers in 2023 during the study period: 3 October (Tom Cameron, TC; 10:40 to 15:40 BST), 4 October (TC; 15:40 to 17:41 BST), 8 October (TC; 12:00 to 12:28 BST), 15 October (TC; 14:54 to 16:59 BST), 16 October 2023 (Edward Codling, EC; 10:30 to 13:03 BST), 23 October (EC; 10:42 to 13:41 BST), 27 October (EC; 13:31 to 16:27 BST), and 3 November (EC; 10:35 to 13:11 GMT). Each collared cow was identified during observations through the color code on their collar (“solid red”, “solid black”, “red and black stripes”). Due to the relatively close proximity, it was possible to continuously observe all three collared cows during each session, except on 16 October 2023 where only cattle ID 294364 was monitored. Observers flagged significant behavioral changes, such as when an individual’s head was raised for over 30 seconds or when grazing was paused. Continuous video recordings were also taken during each session using a digital camera (Sony DCRSR35; records at 3MB per second for up to 20 hours; 40x zoom) to validate observations. The camera was placed on a mobile tripod to ensure it was stable and remained in view of a focal cow during each session. Video 1 shows a snippet of a recording taken during an observation session, illustrating grazing behaviour (cattle ID 294361 on 2024-10-04 at 11:52:00). As outlined in the main text (Section 2.2), sensor-recorded activity was measured using an in-built dynamometer, which monitored movements along the heave axis and totaled them every 30 minutes to generate an "activity index" (as defined by Aeser et al. (1)).

The relationship between the proportion of time each observer recorded cattle to be grazing and the activity index (measured every 30 minutes by Nofence® sensors) was analysed using a mixed linear model, with observer as a random effect. Observer data was aggregated into 30-minute intervals to calculate the proportion of grazing for each interval. Additionally, cumulative activity distributions using the cell count method (refer to Section 2.4 in main paper) during the observation periods are shown, to check the data correspond to observed grazing activity.

Activity index increases as the proportion of time cattle spent grazing increases for each observer (Fig S2.1 A,C). Both observers recorded 30 minute periods of no grazing as well as periods of only grazing, with higher activity values recorded during the latter (Fig S2.1 B,D). There is no significant difference in the relationships between the proportion of time spent grazing against activity between the two observers (e = -6.13, SE = 4.36, t-value = -1.44, *p* = 0.15). As per the observations, the cattle are shown to only be active in the upper field during half of the observation periods (Fig S2.2 D-G) and spread across the upper and lower field during the remaining observation periods (Fig S2.2 A-C,F).

**Fig S2.1.** (A,C) Activity index in relation to proportion of time spent grazing and (B,D) comparison of activity during non-grazing periods (proportion of time spent grazing = 0%) and grazing periods (proportion of time spent grazing = 100%). Data in (A-B) were collected by TC and data in (C-D) were collected by EC. Each data point corresponds to a 30-minute observation period (and associated sensor activity index measurement).

**Fig S2.2.** Cumulative activity distribution maps generated from the cell count method across all three cattle according to observation period: (A) 3 October 2023 (10:40 to 15:40, n = 37 data points), (B) 4 October 2023 (15:40 to 17:41, n = 31 data points), (C) 8 October 2023 (12:00 to 12:28, n = 8 data points), (D) 15 October 2023 (14:54 to 16:59; n = 32 data points), (E) 16 October 2023 (10:30 to 13:03; n = 17 data points), (F) 23/10/2023 (10:42 to 13:41; n = 38 data points), (G) 27 October 2023 (13:31 to 16:27; n = 52 data points) and (H) 3 November 2023 (10:35 to 13:11, n = 43 data points). Data in (A-D) were collected by TC and data in (E-H) were collected by EC. Data were collected from three individuals (IDs 294322, 294361 and 294364) for each observation period except for on 16/10/2023 (E) where data were collected only from individual ID 294364. Each virtual cell is 225 m^2^ and darker colors correspond to higher activity whereas lighter colors correspond to lower activity and contours show the core range size (50%; solid grey) and the full range size (95%; dashed grey). The red dashed line is the contour of the ridge line dividing the upper field and the lower field.

**References**

1. Aaser MF, Staahltoft SK, Andersen M, Alstrup AKO, Sonne C, Bruhn D, Frikke J, Pertoldi C. Using Activity Measures and GNSS Data from a Virtual Fencing System to Assess Habitat Preference and Habitat Utilisation Patterns in Cattle. *Animals* (2024) 14:1506. doi: 10.3390/ani14101506
