# Supplementary material for: Mapping activity of grazing cattle using commercial virtual fencing technology
Source: Front Vet Sci. 2025 Mar 12;12:1536977. doi: 10.3389/fvets.2025.1536977 (PMC11960750; doi:10.3389/fvets.2025.1536977)
Supplement: Supplementary file 3 [file Data_Sheet_3.DOCX]

Supplementary Material 3: Distribution comparisons

Here, we present detailed explanations of the cell count method and Brownian Bridge Movement Model (BBMM) used to generate distribution (density) maps of space-use (Section 1.1 for cell count method and Section 2.1 for the BBMM), cumulative activity and average activity (Section 1.2 for cell count method and Section 2.2 for the BB2MM), and temperature (Section 1.3). We also show additional distribution maps, for space-use, cumulative activity, and average activity, along with comparisons for each individual across the entire study period (Section 3.1), according to time of day across all cows (Section 3.2) and for each two-week period (fortnight) (Section 3.3) using both methods.

# Cell count method

## Space-use (cell count)

Space-use (utility) distribution maps were generated by overlaying a virtual grid (15m x 15m = 225m^2^ cells) over the field. At each time-step, GPS data were used to assign each individual cow to a given virtual cell, and the respective cell count value was increased by the duration until the next location recording (typically 15 minutes). The count was continued across the three cows to give a final total time spent in each virtual cell within the field.

## Activity (cell count)

In general, activity index values were recorded every 30 minutes while GPS locations were recorded every 15 minutes (approximately one minute apart) (1). To determine the cumulative activity distribution, the total activity for each 30-minute period was halved and assigned equally to the two most recent recorded locations. Where data were recorded at different frequencies (i.e., during periods of inactivity), activity data were assigned proportionally. A 15m x 15m virtual grid was overlayed over the field and at each time-step location data were again used to assign each individual to a given cell. However, the cell count value was increased by the respective activity value (rather than time duration). This count was continued across the three cows to give a final total cumulative activity for each virtual cell within the field.

To compute the average activity distribution, the cumulative activity in each cell was divided by the total time spent in that cell.

## Temperature (cell count)

Instantaneous temperature readings were recorded by the Nofence® sensor every 30 minutes, approximately one minute after the most recent location recording. To plot average temperature distribution maps, the same 15m x 15m virtual grid was overlayed over the field layout and at each 30-minute time-step location data were again used to assign each individual to a given cell. The corresponding temperature values were assigned to each cell, summed, and divided by the number of location points assigned to that cell in the given time period, to give a final average temperature value for each virtual cell within the field over the relevant time period.

# Brownian Bridge Method

## Space-use (BBMM)

A BBMM (2) was used to estimate utility distribution (UD). By adapting the R function created in Sawyer et al. (3) (see Supplement 1 in their study) a standard BBMM was used where the probability of an individual being present in an area is based on distance, time between consecutive fixes and a five meter location error (average GPS sensor accuracy, 4–6). The underlying virtual grid size was set to 15m x 15m (225m^2^ cells), consistent with the cell count method above. The estimate of an individual’s mobility, A Brownian Movement Variance (BMV) (2) is usually estimated across the whole movement path of the animal [as in 87] but can be inaccurate with few steps, and would affect step-by-step activity estimates we use in the next section. While a dynamic BBMM could account for varying movement, where BMV varies over a path, could account for heterogenous movement i.e., as in (3) and (7), a fixed BMV was used due to the small field area and the consistent movement behaviour observed. The BMV was set to 70, based on the average for each individual and day of the study when applying a BBMM to the full dataset. The time integration step in the BBMM was set as one minute considering the typical movements of cattle. A new UD was therefore generated for each two consecutive locations, with a sum of the estimated time duration for the three individuals (or a singular individual) allocated to each cell.

## Activity (BBMM)

A simplifying assumption was made by equally dividing the total activity for each 30-minute period between the two most recently recorded 15-minute intervals and their locations (or adjusted proportionally for occasionally differing frequencies). To determine a cumulative activity distribution, the BBMM was implemented step-by step, creating a new space-use distribution for each 15-minute period, unlike the usual implementation of the BBMM that produces a single distribution from the entire time series (3). The density of each virtual cell for a specific period was multiplied by the assigned activity value for that 15-minute period, allocating activity across space relative to the estimated location within the BBMM. This process was continued step-by-step for the full dataset, resulting in a final cumulative activity distribution by cumulatively totaling each stepwise distribution previously determined. Finally, an average activity distribution was determined by dividing the total BBMM cumulative activity for a given cell by the respective time duration (calculated for the BBMM space-use distribution) for that cell over the appropriate period.

# Additional distribution comparisons

The fortnightly divisions are defined as follows: F1 = 29 September 2023 to 13 October 2023, F2 = 14 October 2023 to 28 October 2023, F3 = 29 October 2023 to 12 November 2023 and F4 = 13 November 2023 to 26 November 2023 (note that F4 includes 15 days). Times of the day are divided over the full study as: night (00:00:00 to 05:59:59), morning (06:00:00 to 11:59:59), afternoon (12:00:00 to 17:59:59) and evening (18:00:00 to 23:59:59). We also show the results of comparisons between temperature distribution maps by fortnight and time of day (following Section 2.4 in main paper).

Distribution comparisons were conducted using the Bhattacharya coefficient (BC), which provides a single measure of similarity between two distributions (refer to Section 2.4.1 in main paper). However, it is important to note that determining whether these similarities are statistically significant would require a randomization test, such as the one used in Chopra et al. (8). Due to the limited sample size in this study, significance tests comparing BC were not conducted. Therefore, our comparisons are intended to provide illustrative examples rather than definitive conclusions.

## Full study period

Although a full statistical analysis of individual differences is not sensible due to the limited sample size (n = 3 cows), each individual cow is observed to have qualitatively similar behaviour (i.e. comparing Figs S3.1A, S3.1D, S3.1G and similar for S3.1B, S3.1E, S3.1H and S3.1.C, S3.1F, S3.1I and similar for Fig S3.2; BC ranges from 0.88 to 0.90 for cell count method and BC is consistently 0.99 for BBMM), albeit some minor variations. Strong overlap is also shown between the individual cumulative activity distributions (BC ranges from 0.94 to 0.95 for cell count method and BC ranges from 0.98 to 0.99 for BBMM), and average activity distributions (BC ranges from 0.92 to 0.93 for cell count method and BC consistently ≥ 0.99 for BBMM) (Table S3.1; refer to Fig S3.1-2). However, some subtle differences are visible between individual cattle (e.g., compare the core range isopleths in the lower SE corner of the field in Figs. S3.1A, 2.1D, 2.1G); the largest difference in space-use was between cattle ID 29322 and cattle 294361 (Figs S3.1A, S3.1D, BC = 0.88) for the cell count method (although space-use similarity for the BBMM was still high, Figs S3.2A, S3.2D, BC = 0.99).

Space-use is highly correlated with cumulative activity for each individual (BC = 0.93, 0.95 and 0.94 for cattle ID 29322, cattle 294361 and cattle 294364 respectively according to cell count method, Fig S3.1B, S3.1E, S3.1H; BC consistently 0.99 for BBMM, Fig S3.2B, S3.2E, S3.2H). Furthermore, average activity intensity does not always overlap directly with cumulative activity for each individual (BC = 0.93, 0.92 and 0.92 for cattle ID 29322, cattle 294361 and cattle 294364 respectively according to cell count method, BC = 0.93, 0.93 and 0.94 for cattle ID 29322, cattle 294361 and cattle 294364 respectively according to BBMM) (see Fig S3.1).

**Figure S3.1.** Distribution maps generated using the cell count method: (A,D,G) space-use, (B,E,H) cumulative activity and (C,F,I) average activity distributions for each individual: (A-C) show cattle ID 29322 (n = 4250 data points), (D-F) show cattle ID 294361 (n = 5570 data points) and (G-I) show cattle ID 294364 (n = 5508 data points). Each virtual cell is 225 m^2^ and darker colours correspond to higher activity whereas lighter colours correspond to lower activity and contours show the core range size (50%; solid grey) and the full range size (95%; dashed grey). The red dashed line is the contour of the ridge line dividing the upper field and the lower field.

**Figure S3.2.** Distribution maps generated using the BBMM method: (A,D,G) space-use, (B,E,H) cumulative activity and (C,F,I) average activity distributions for each individual: (A-C) show cattle ID 29322 (n = 4250 data points), (D-F) show cattle ID 294361 (n = 5570 data points) and (G-I) show cattle ID 294364 (n = 5508 data points). Each virtual cell is 225 m^2^ and darker colours correspond to higher activity whereas lighter colours correspond to lower activity and contours show the core range size (50%; solid grey) and the full range size (95%; dashed grey). The red dashed line is the contour of the ridge line dividing the upper field and the lower field.

**Table S3.1.** Individual distribution comparisons for space-use, cumulative activity, and average activity. Three comparisons are drawn between cattle IDs 294322, 294364 and 294361. Bhattacharya coefficients are shown (BC = 0 indicating complete dissimilarity whereas 1 indicating complete overlap) for comparisons of distributions generated from a cell count method and a BMM.

| **Cell count method** | | | **BBMM** | | |  |
| --- | --- | --- | --- | --- | --- | --- |
| **Cattle IDs** | **Space-use** | **Cumulative activity** | **Average activity** | **Space-use** | **Cumulative activity** | **Average activity** |
| 294322- 294361 | 0.88 | 0.95 | 0.93 | 0.99 | 0.99 | > 0.99 |
| 294322- 294364 | 0.89 | 0.94 | 0.92 | 0.99 | 0.98 | 0.99 |
| 294361- 294364 | 0.90 | 0.95 | 0.92 | 0.99 | 0.99 | 0.99 |

## Daily patterns (time of day)

A clear diurnal pattern in space-use is apparent in both models. The cattle spent the evening and night closer to the ridgeline and lower field (Fig S3.3A, core range size at night in upper field = 14 cells for cell count method; Fig S3.4A, core range size at night in upper field = 28 cells for BBMM; Fig S3.3D, core range size in evening in upper field = 15 cells for cell count method; Fig 7D, core range size in evening in upper field = 29 cells for BBMM) before shifting their activity to the upper field in the morning and afternoon, with some movement back below the ridgeline towards late afternoon (Fig S3.3B, core range size in morning in upper field = 58 cells for cell count method; Fig S3.4A, core range size in morning in upper field = 78 cells for BBMM; Fig S3.3C, core range size in afternoon in upper field = 61 cells for cell count method; Fig S3.4C, core range size in afternoon in upper field = 71 cells for BBMM). This diurnal pattern is also reflected in the similarity between the space-use distributions e.g., the morning and afternoon are similar (BC = 0.92 for cell count method and BC = 0.96 for BBMM; Table S3.2) whereas the morning distribution shows less overlap with the evening and night distributions (BC = 0.78 for cell count method and BC = 0.94 for BBMM for evening; BC = 0.75 for cell count method and BC = 0.92 for BBMM for night; Table S3.2).

Similarly, the cumulative activity distributions show high overlap between the morning and afternoon (BC = 0.94 for cell count method and 0.96 for BBMM; Table S3.2) and the least overlap between the morning and night (BC = 0.73 for cell count method and 0.82 for BBMM; Table S3.2). These cumulative activity patterns algin with this diurnal shift in activity, with higher activity intensity in the upper field during the morning and afternoon, and limited activity in this area in the evening and night (Figs S3.3E-H, cell count method core range size in upper field = 13 cells at night, 91 cells in morning, 67 cells in afternoon, 15 cells in evening; Figs S3.4E-H, BBMM core range size = 18 cells at night, 85 cells in morning, 66 cells in afternoon, 13 cells in evening), also consistent with the diurnal pattern of activity shown in Fig 2E in the main paper. The core range for the (mean) average activity intensity is also generally higher in the upper field in the morning and afternoon and lower in the evening and night (Figs S3.3I-L, S3.4I-L). However, it is interesting to note that, although the largest core range for the cumulative activity intensity is actually in the upper field in the afternoon (compare Figs S3.3G and S3.3K, S3.4G and S3.4K), there is a larger (relative) core range for average activity in the lower field later in the afternoon even though cows were active in both the upper and lower field areas at these times (Figs S3.3I-L, cell count method core range size in upper field = 27 cells at night, 74 cells in morning, 54 cells in afternoon, 27 cells in evening; Figs S3.4I-L, BBMM core range size = 25 cells at night, 96 cells in morning, 42 cells in afternoon, 48 cells in evening). This highlights not only a shift in where the cattle were active throughout the day, but also how intensively they were active these different areas at different times. Note that Fig S3.4L seems to suggest that there is a large core range for average activity intensity in the upper field in the evening, although this may be an artifact of the BBMM and should not be over-interpreted given the relatively low number of data points for this area at this time (compare to Fig S3.3L).


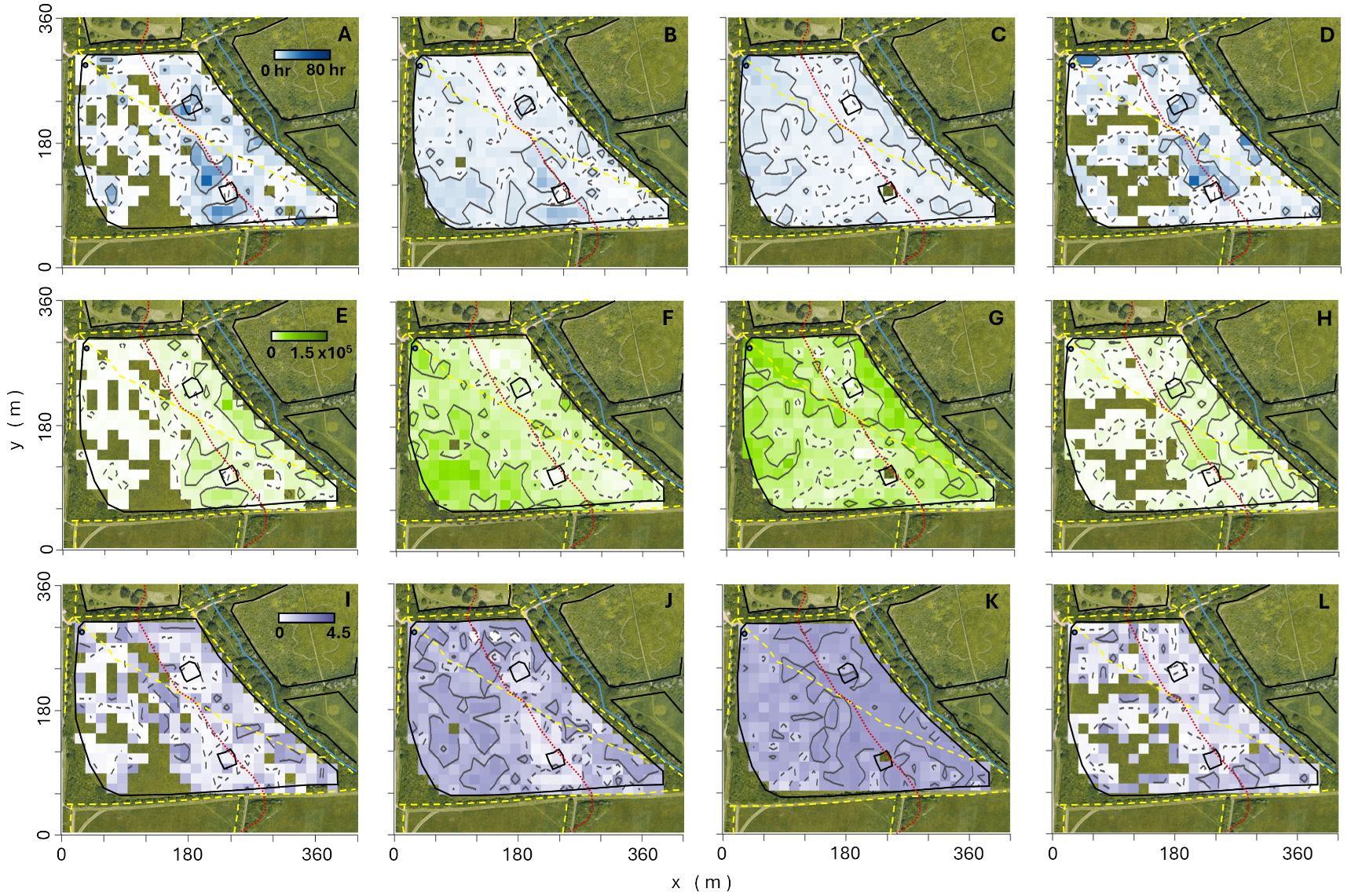


**Fig S3.3.** Time of day distribution maps across all three cattle generated using the cell count method: (A-D) Space-use, (E-H) cumulative activity and (I-L) average activity: (A, E,I) night (00:00:00 to 05:59:59; n = 2765 data points), (B, F,J) morning (06:00:00 to 11:59:59; n = 4519 data points), (C,G,K) afternoon (12:00:00 to 17:59:59; n = 5156 data points) and (D,H,L) evening (18:00:00 to 23:59:59; n = 2888 data points). Each virtual cell is 225 m^2^. Note that cells showing the satellite underlay are empty (no data recorded). The red dashed line is the contour of the ridge line dividing the upper field and the lower field.


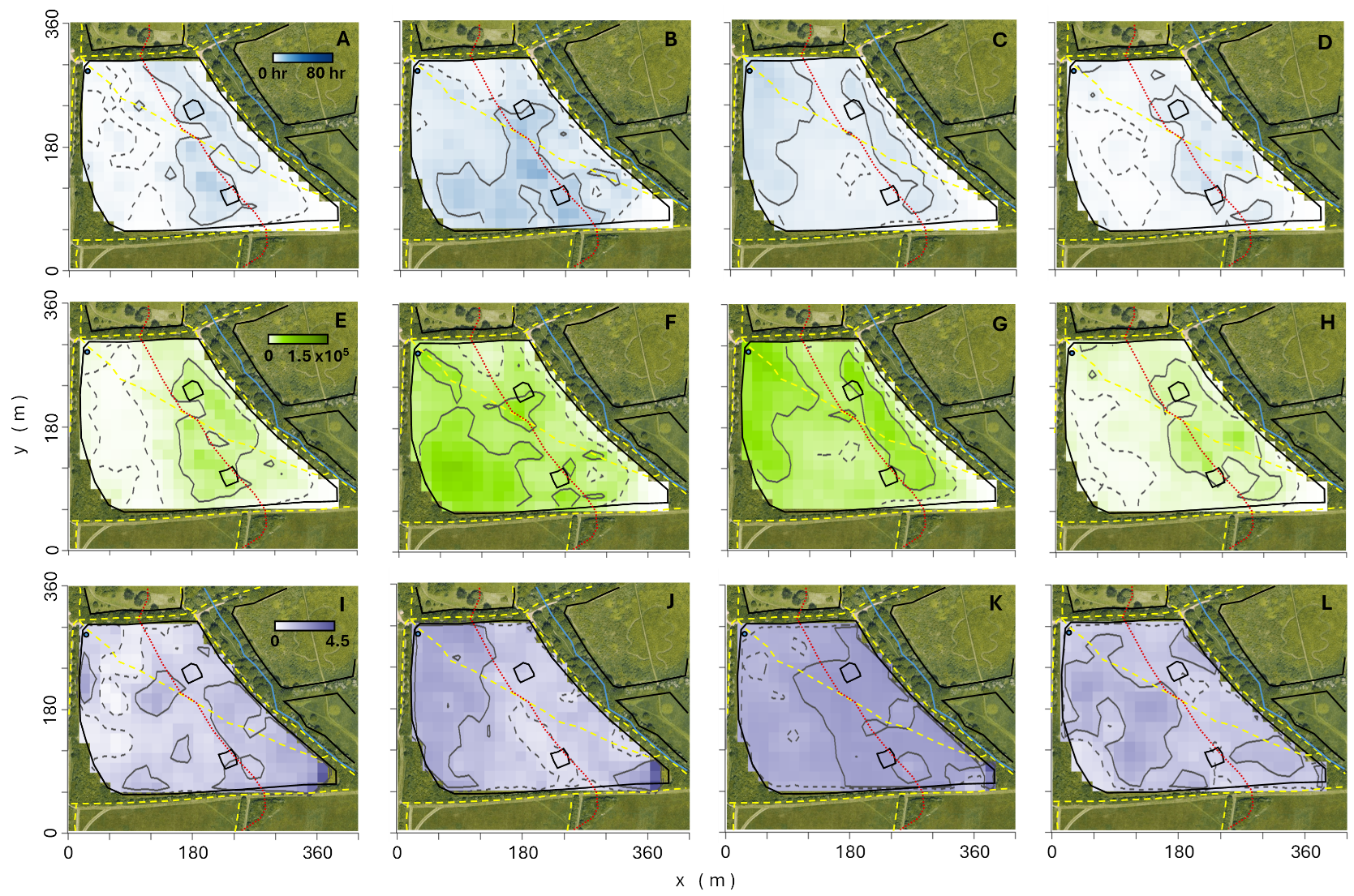


**Fig S3.4.** Time of day distribution maps across all three cattle generated using the BBMM method: (A-D) Space-use, (E-H) cumulative activity and (I-L) average activity: (A, E,I) night (00:00:00 to 05:59:59; n = 2765 data points), (B, F,J) morning (06:00:00 to 11:59:59; n = 4519 data points), (C,G,K) afternoon (12:00:00 to 17:59:59; n = 5156 data points) and (D,H,L) evening (18:00:00 to 23:59:59; n = 2888 data points). Each virtual cell is 225 m^2^. Note that cells showing the satellite underlay are empty (no data recorded). The red dashed line is the contour of the ridge line dividing the upper field and the lower field.

**Table S3.2.** Time of day distribution comparisons for space-use, cumulative activity, and average activity. Time of day is split into night (00:00:00 to 05:59:59; N), morning (06:00:00 to 11:59:59; M), afternoon (12:00:00 to 17:59:59; A) and evening (18:00:00 to 23:59:59; E). Bhattacharya coefficients are shown (BC = 0 indicating complete dissimilarity whereas 1 indicating complete overlap) for comparisons of distributions generated from a cell count method and a BBMM.

| **Cell count method** | | | | **BBMM** | | | |
| --- | --- | --- | --- | --- | --- | --- | --- |
| **Time of day comparison** | **Space-use** | **Cumulative activity** | **Average activity** | **Space-use** | | **Cumulative activity** | **Average activity** |
| N-M | 0.78 | 0.73 | 0.77 | 0.94 | | 0.82 | 0.92 |
| N-A | 0.70 | 0.79 | 0.79 | 0.89 | | 0.89 | 0.96 |
| N-E | 0.79 | 0.84 | 0.71 | 0.96 | | 0.96 | 0.97 |
| M-A | 0.92 | 0.94 | 0.94 | 0.96 | | 0.97 | 0.97 |
| M-E | 0.75 | 0.76 | 0.77 | 0.92 | | 0.88 | 0.96 |
| A-E | 0.75 | 0.83 | 0.82 | 0.92 | | 0.94 | 0.99 |

## Longer-term changes (fortnights)

Both models show a clear and consistent trend over the fortnights of the study (F1-F4), with the upper field being used less and the lower field used more as the study progressed (Figs S3.5A-D, core range size in upper field for cell count method = 43 cells for F1, 33 cells for F2, 9 cells for F3, 5 cells for F4; Figs S3.6A-D, core range size in upper field for BBMM = 76 cells for F1, 80 cells for F2, 42 cells for F3, 14 cells for F4). This change over time is also apparent in the cumulative activity with a shift later in the study towards the lower field (Figs S3.5E-H, core range size in lower field for cell count method = 48 cells for F1, 16 cells for F2, 51 cells for F3, 67 cells for F4; Figs S3.6E-H, core range size in lower field for BBMM = 44 cells for F1, 10 cells for F2, 39 cells for F3, 61 cells for F4). Over the same time period, the core range for the (mean) average activity increases in the upper field area (Figs S3.5I-L, core range size in upper field for cell count method = 62 cells for F1, 83 cells for F2, 78 cells for F3, 86 cells for F4; Figs S3.6E-H, core range size in upper field for BBMM = 40 cells for F1, 98 cells for F2, 96 cells for F3, 85 cells for F4) indicating that although the cattle are spending less time in the upper field area as the study progresses, they seem to be (relatively) more active when they were active there (which matches with Figs 2C,D,E). This shift over the study is also reflected in comparisons of the distributions, with the most overlap generally shown between consecutive fortnights e.g., for space-use for F1 and F2, BC = 0.83 for cell count method and BC = 0.97 for BBMM whereas for F1 and F4, BC = 0.76 for the cell count method and BC = 0.92 for BBMM (Table S3.3; Figs S3.5-6).


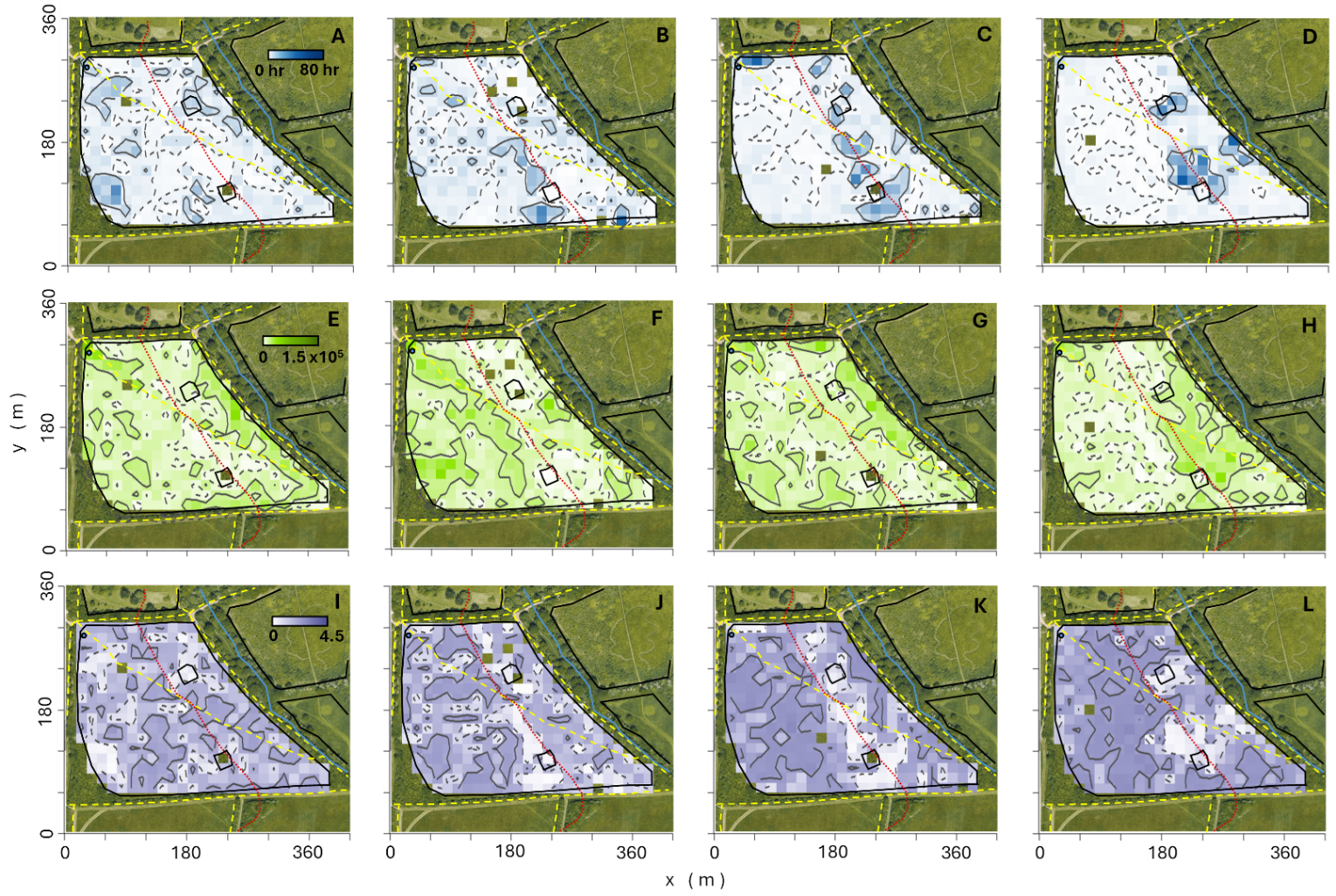


**Fig S3.5.** Fortnightly distribution maps across all three cattle generated using the cell count method: (A-D) Space-use, (E-H) cumulative activity and (I-L) average activity across the herd (note that fortnight (F) 1 = 15 days). (A,E,I) F1 = 29 September 2023 to 13 October 2023 (n = 4053 data points) (B,F,J) F2 = 14 October 2023 to 28 October 2023 (n = 3943 data points), (C,G,K) F3 = 29 October 2023 to 12 November 2023 (n = 3804 data points) and (D,H,L) F4 = 13 November 2023 to 26 November 2023 (15 days; n = 3528 data points). Each virtual cell is 225m^2^. Note that cells showing the satellite underlay are empty (no data recorded). The red dashed line is the contour of the ridge line dividing the upper field and the lower field.


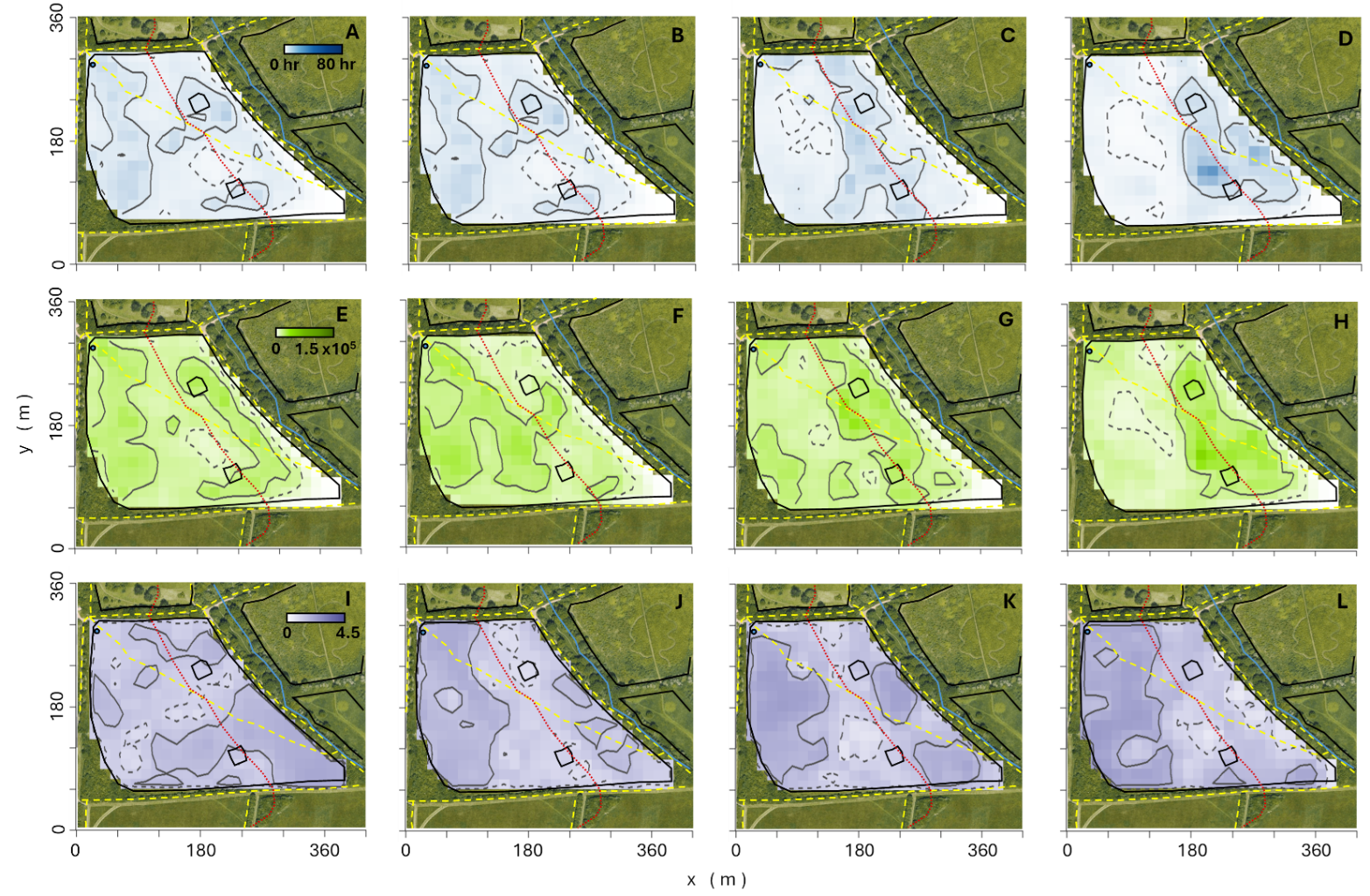


**Fig S3.6.** Fortnightly distribution maps across all three cattle generated using the BBMM method: (A-D) Space-use, (E-H) cumulative activity and (I-L) average activity across the herd (note that fortnight (F) 1 = 15 days). (A,E,I) F1 = 29 September 2023 to 13 October 2023 (n = 4053 data points) (B,F,J) F2 = 14 October 2023 to 28 October 2023 (n = 3943 data points), (C,G,K) F3 = 29 October 2023 to 12 November 2023 (n = 3804 data points) and (D,H,L) F4 = 13 November 2023 to 26 November 2023 (15 days; n = 3528 data points). Each virtual cell is 225m^2^. Note that cells showing the satellite underlay are empty (no data recorded). The red dashed line is the contour of the ridge line dividing the upper field and the lower field.

**Table S3.3.** Comparison of fortnightly distributions for space-use, cumhorneulative activity, and average activity. Fortnights are split as fortnight (F)1 = 29 September 2023 to 13 October 2023, F2 = 14 October 2023 to 28 October 2023, F3 = 29 October 2023 to 12 November 2023 and F4 = 13 November 2023 to 26 November 2023 (15 days). Bhattacharya coefficients are shown (BC = 0 indicating complete dissimilarity whereas BC = 1 indicating complete overlap) for comparisons of distributions generated from a cell count method and a BBMM.

| **Cell count method** | | | | **BBMM** | | | |
| --- | --- | --- | --- | --- | --- | --- | --- |
| **Fortnightly comparison** | **Space-use** | **Cumulative activity** | **Average activity** | **Space-use** | | **Cumulative activity** | **Average activity** |
| F1-F2 | 0.83 | 0.93 | 0.90 | 0.97 | | 0.98 | 0.98 |
| F1-F3 | 0.81 | 0.93 | 0.90 | 0.89 | | 0.99 | 0.93 |
| F1-F4 | 0.76 | 0.92 | 0.91 | 0.92 | | 0.98 | 0.94 |
| F2-F3 | 0.83 | 0.93 | 0.91 | 0.90 | | 0.98 | 0.95 |
| F2-F4 | 0.75 | 0.90 | 0.91 | 0.94 | | 0.96 | 0.94 |
| F3-F4 | 0.82 | 0.93 | 0.93 | 0.96 | | 0.98 | 0.91 |

## Temperature

The temperature distributions during the morning and afternoon show the most overlap (BC = 0.97) compared to the remaining distributions (morning and night: BC = 0.83, morning and evening: BC = 0.84, afternoon and evening: BC = 0.84, afternoon and night: BC = 0.82, evening and night: BC = 0.81) (see Fig 4 in main paper). Furthermore, the fortnightly temperature distributions highly overlap (BC = ranging from 0.90 to 0.95 for each of the six fortnightly comparisons) (Table S3.4; refer to Fig 4 in main paper).

Table S3.4. Temperature distribution comparisons between fortnights across the three cattle. Fortnights are split as fortnight (F)1 = 29 September 2023 to 13 October 2023, F2 = 14 October 2023 to 28 October 2023, F3 = 29 October 2023 to 12 November 2023 and F4 = 13 November 2023 to 26 November 2023 (15 days). Bhattacharya coefficients are shown (BC = 0 indicating complete dissimilarity whereas 1 indicating complete overlap).

| **Fortnight** | F1 | F2 | F3 | F4 |
| --- | --- | --- | --- | --- |
| F1 | - | 0.95 | 0.93 | 0.91 |
| F2 | 0.95 | - | 0.94 | 0.91 |
| F3 | 0.93 | 0.94 | - | 0.90 |
| F4 | 0.91 | 0.91 | 0.90 | - |

**References**

1. Aaser MF, Staahltoft SK, Andersen M, Alstrup AKO, Sonne C, Bruhn D, Frikke J, Pertoldi C. Using Activity Measures and GNSS Data from a Virtual Fencing System to Assess Habitat Preference and Habitat Utilisation Patterns in Cattle. *Animals* (2024) 14:1506. doi: 10.3390/ani14101506

2. Horne JS, Garton EO, Krone SM, Lewis JS. Analyzing Animal Movements Using Brownian Bridges. *Ecology* (2007) 88:2354–2363. doi: 10.1890/06-0957.1

3. Sawyer H, Kauffman MJ, Nielson RM, Horne JS. Identifying and prioritizing ungulate migration routes for landscape-level conservation. *Ecol Appl* (2009) 19:2016–2025. doi: 10.1890/08-2034.1

4. D’Eon RG, Serrouya R, Smith G, Kochanny CO. GPS Radiotelemetry Error and Bias in Mountainous Terrain. *Wildl Soc Bull 1973-2006* (2002) 30:430–439.

5. Frair JL, Fieberg J, Hebblewhite M, Cagnacci F, DeCesare NJ, Pedrotti L. Resolving issues of imprecise and habitat-biased locations in ecological analyses using GPS telemetry data. *Philos Trans R Soc B Biol Sci* (2010) 365:2187–2200. doi: 10.1098/rstb.2010.0084

6. Nofence. Nofence. (2024) https://www.nofence.no/en-gb [Accessed June 29, 2024]

7. Kranstauber B, Kays R, LaPoint SD, Wikelski M, Safi K. A dynamic Brownian bridge movement model to estimate utilization distributions for heterogeneous animal movement. *J Anim Ecol* (2012) 81:738–746. doi: 10.1111/j.1365-2656.2012.01955.x

8. Chopra K, Hodges HR, Barker ZE, Diosdado JAV, Amory JR, Cameron TC, Croft DP, Bell NJ, Thurman A, Bartlett D, et al. Bunching behavior in housed dairy cows at higher ambient temperatures. *J Dairy Sci* (2024) 107:2406–2425. doi: 10.3168/jds.2023-23931
